# Conjunctival geographic ulcer and blepharitis in primary ocular herpes: a case report

**DOI:** 10.4076/1757-1626-2-8141

**Published:** 2009-07-29

**Authors:** Rohina Swaroop

**Affiliations:** 3501 Champion Lake Boulevard, #1609, Shreveport, LA 71105USA

## Abstract

**Introduction:**

Conjunctival geographic ulcer is a rare manifestation of ocular herpes simplex. Geographic ulcers are formed when sloughing of the epithelium occurs in the areas between the dendrite and a broad area of epithelial involvement with irregular angulated borders is formed.

**Case presentation:**

We report a case of primary ocular herpes with blepharitis and geographic ulceration of the conjunctiva in an 8-year-old male with no corneal lesion. To the best of our knowledge only 4 cases of conjunctival herpetic dendritic ulcerations and a single case of herpetic geographical ulcer have been reported in literature till date.

**Conclusion:**

This is a rare clinical presentation of primary ocular herpes and ophthalmologists need to be aware and vigilant of this, as one of the myriad manifestations of primary ocular herpes, thereby providing appropriate therapy/treatment.

## Introduction

HSV infection is the most common cause of blindness in developed countries. Primary infection with HSV-1 is silent in the majority of cases, with as few as 6% of cases being symptomatic .The lesions develop 10-12 days after exposure to the virus. Primary ocular herpes usually presents as blepharitis, blepharoconjunctivitis or keratitis. Conjunctival ulceration in the form of dendrites or geographic ulcers in ocular herpes is very rare.

In a study conducted in Rochester, Minnesota [1] over a period from 1950 to 1982 it was observed that Primary ocular herpes involved lid or conjunctiva in 54%, superficial cornea in 63%, deeper cornea in 6%, and uveitis in 4%. Darouger et al [2] in a study conducted in 108 patients of primary ocular herpes found 14 (12.9%) patients to be in the age group 5-10 years of age.

Herpes simplex virus infections [3] involving the lid may present in one of two forms. The classic appearance involves an accumulation of small vesicles or pustules along the lid margin and/or periocular skin, itching may be associated symptom. These lesions have an inflamed, erythematous base. Within the first week of infection, the vesicles may ulcerate or harden into crusts. Once the crusts fall off the lesion is no longer infectious. Erosive-ulcerative form of HSV blepharitis has also been described. This presentation is characterized by erosions of the lid at the Gray line or ulcers along the lid margin, or a combination of both. Spread of the virus from eyelids and conjunctiva to cornea is uncommon in children even without antiviral prophylaxis. A study conducted in Japan [4] even suggested that a biological difference may exist between HSV strains causing keratitis and conjunctivitis.

Conjunctival geographic ulcer is a rare manifestation of ocular herpes simplex. Geographic ulcers are formed when sloughing of the epithelium occurs in the areas between the dendrite and broad area of epithelial involvement with irregular angulated borders is formed. To the best of our knowledge 4 cases of conjunctival dendritic ulcerations [5,6] and [7] a single case of herpetic geographical ulcer has been reported in literature till date. Only 1 of the reported cases of conjunctival dendrite had primary ocular herpes.

The reasons for herpetic lesions having a dendritiform and on progression of the disease, a geographic pattern are not known. Earlier it was postulated that the shape was due to herpes involving the corneal nerves. Another school of thought [8] was that it was due to “polygonal cells” in epithelium. The demonstration of dendrites on the conjunctiva refutes both these theories.

## Case presentation

A 8-year-old Asian Indian male presented with complaints of pain, swelling and eruptions on right upper and lower lids for the last 7 days. No significant past medical/surgical/ family/social history was elicited. No rhinitis/pharyngitis/fever was present. No preauricular lymphadenopathy was present. Vision was 6/6 in both eyes. Vesicular eruptions were present on right side of face near the periorbital region, and right upper and lower lids showed swelling and vesicular eruption. Corneal sensations were diminished in the right eye.

Slit lamp examination of the right eye with fluorescein staining showed well defined area of fluorescein staining in the lower bulbar conjunctiva resembling a geographic ulcer with irregular, angulated margins. Anterior segment and posterior segment examination were unremarkable. Patient was started on Acyclovir (3%) ointment 5 times/day local application on eye and skin and oral Acyclovir 200 mg 5 times/day. He was reviewed after 3 days and then after 12 days. Patient recovered completely after 12 days and medications were terminated.

## Conclusion

This is a rare clinical presentation of primary ocular herpes and ophthalmologists need to be aware and vigilant of this, as one of the myriad manifestations of primary ocular herpes, thereby providing appropriate therapy/treatment.

**Figure 1.**
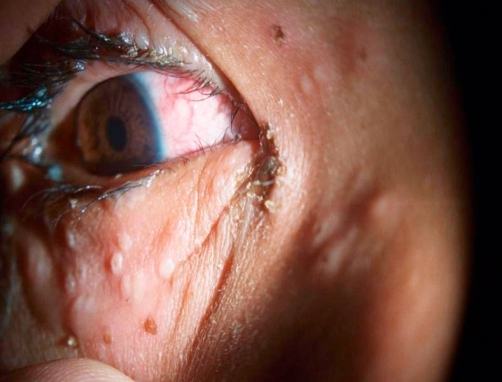
Eruptions in the Right periorbital region. Small vesicles can be seen along the lid margin and periocular skin. Figure also shows crusting of the eyelids and conjunctival congestion.

**Figure 2.**
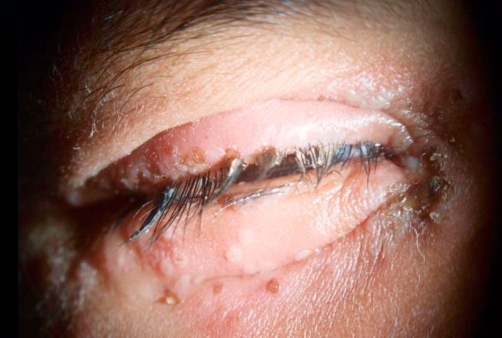
Herpetic blepharitis. Swelling and erythema of the eyelids along with erosions and ulcerations of the lid margins can be seen.

**Figure 3.**
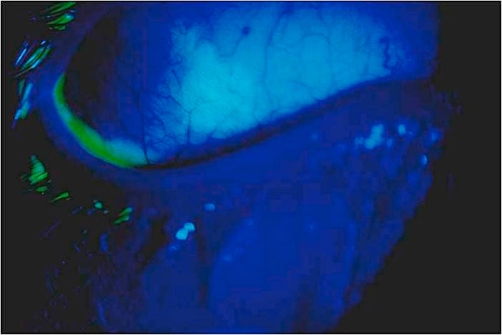
Fluorescein staining in the lower bulbar conjunctiva. Well defined geographic ulcer with irregular, angular, dendritiform margins can be seen on fluorescein staining of the lower bulbar conjunctiva.

**Figure 4.**
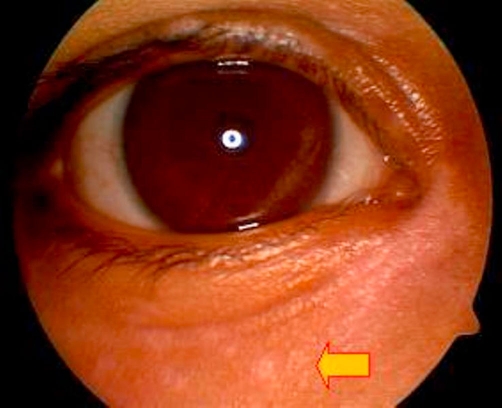
Healed blepharitis and periorbital skin lesions after 12 days of therapy. Only slight hypopigmentation at the site of the previous skin lesions can be observed after 12 days of therapy.

**Figure 5.**
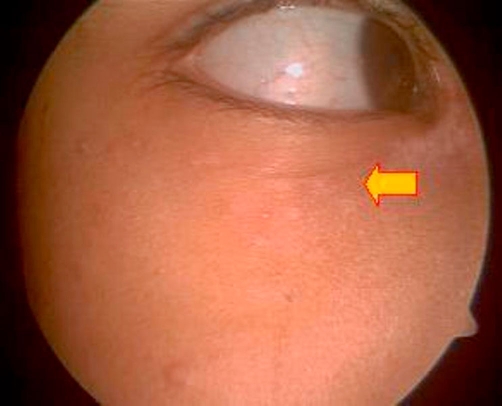
Healed blepharitis and periorbital skin lesions after 12 days of therapy. Only slight hypopigmentation at the site of the previous skin lesions can be observed after 12 days of therapy.

**Figure 6.**
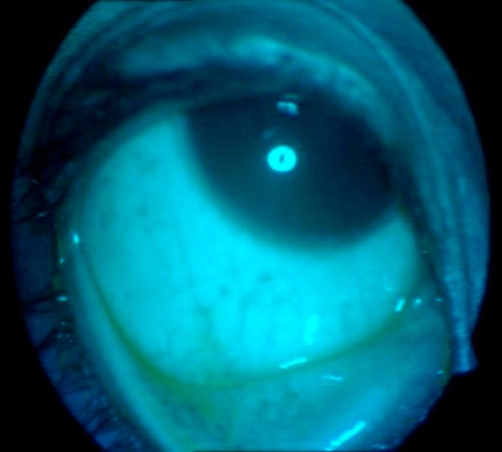
Healed conjunctival geographic ulcer after 12 days of therapy. No fluorescein staining in the lower bulbar conjunctiva after 12 days of therapy. Geographic ulcer has completely healed.

## Abbreviations

HSV: Herpes Simplex Virus
HSV-1: Herpes Simplex Virus type 1
